# GLA insufficiency should not be called Fabry disease

**DOI:** 10.1038/s41431-024-01657-0

**Published:** 2024-06-27

**Authors:** Gunnar Houge, Mirjam Langeveld, Joao-Paulo Oliveira

**Affiliations:** 1 Department of Medical Genetics, Haukeland University Hospital, Bergen, Norway; 2 Institute of Clinical Medicine K2, Faculty of Medicine, University of Bergen, Bergen, Norway; 3 Department of Endocrinology and Metabolism, Amsterdam UMC, Research Institute of Gastroenterology, Endocrinology & Metabolism (AGEM), University of Amsterdam, Amsterdam, The Netherlands; 4 Service of Medical Genetics, São João University Hospital Center, Porto, Portugal; 5 Department of Pathology, Unit of Genetics, University of Porto, Porto, Portugal

**Keywords:** Metabolic disorders, Risk factors, Prognostic markers, Medical genetics

There is a tendency to make a clinical genetic diagnosis just based on the finding of an assumed pathogenic variant in a causative gene or a chromosome finding, analogous to trisomy 21 equals Down syndrome. Such a short cut is rarely warranted even though the diagnosis later may prove to be correct. Clinical diagnoses should be made on clinical grounds, based on symptoms and findings. Maybe a diagnosis is supported or suggested by genetic findings, but the genetic finding rarely equals the diagnosis in an index patient. Notable exceptions are high-penetrant genetic variants that are so well known that the clinical diagnosis is a given either at present or in the future.

Regarding the X-linked metabolic disorder Fabry disease, this genotype-syndrome shortcut is particularly problematic since an inappropriate Fabry diagnosis may lead to unnecessary use of expensive enzyme replacement or substrate reduction therapies, which is then perceived to be effective since no further symptoms develop or the patient feels better. Moreover, patients may think they have a monogenic syndrome (Fabry disease) while instead they have a multifactorial disease with low GLA activity as one of several potentially contributing factors.

Classic Fabry disease is caused by severe alpha-galactosidase A (GLA) deficiency, has a multi-organ phenotype, and the enzyme activity is abolished or close to zero. The main cause is loss-of-function (LoF) variants in the X-linked *GLA* gene. Both males and females are affected, and in females the degree of disease is thought to depend on the X-inactivation pattern, which in most cases is random. So-called non-classic Fabry disease, associated with incomplete GLA deficiency, is usually caused by missense changes in the *GLA* gene that lowers but do not abolish enzyme activity. The residual activity can vary significantly between variants, which is reflected by the great variation in the rise of substrate levels as well as clinical manifestations, which are frequently confined to the heart. This entity should not be called non-classical Fabry disease but GLA insufficiency, which denotes incomplete GLA deficiency. For many patients and doctors, the term “non-classical Fabry disease” still means “Fabry disease” – we therefore believe GLA insufficiency is a better term.

Upon genetic testing of *GLA*, sometimes the result is a clear-cut loss-of-function (LoF) variant (e.g., deletions, nonsense variants, frameshifting and consensus splice site variants), in other cases not. The latter concerns many missense variants, i.e., variants that predict an amino acid exchange. *GLA* is a gene with much missense variation, reflected by a gnomAD 2.1.1. database z-score of 1.88. [1] Some missense variants cause Fabry disease because they abolish enzymatic function, just like a LoF variant. Others just lower enzymatic activity, sometimes to just few percent of normal level. We call them low penetrant and hypomorphic alleles, a term implying that such variants rarely or never cause disease by themselves. This concerns a large group of variants, many published as causative for Fabry disease, especially in older literature. More recently, ER stress triggering an unfolded protein response has been suggested as an alternative disease mechanism for some *GLA* missense variants, but since the evidence so far is based on variant overexpression in cell lines, it is so far unknown if this mechanism has any clinical relevance [2].

There is no defined threshold for when the GLA activity is so low that Fabry disease will develop. The problem with the most widely used assay to measure alpha-galactosidase activity (the 4-MU assay) is that it does not measure accurately in the lower range. Therefore, we believe the best guide for when “low is too low” is not the enzyme activity but the plasma lyso-globotriaosylceramide level (lyso-Gb3 level). High lyso-Gb3 in males or females is a stable indicator of GLA activity in the Fabry-disease range, useful for distinguishing between Fabry disease and GLA insufficiency [3].

Population-frequent missense variants in *GLA* (like Arg112His and Ala143Thr) do not cause classical Fabry disease, [4] but the GLA enzyme activity is reduced. Since these variants are considered as “likely pathogenic” or “pathogenic” in most ClinVar database entries and many articles, and since the only associated diagnosis to low GLA activity is Fabry disease, many variant-carrying individuals are wrongly labeled as “Fabry patients”. Both variants are associated with low lyso-Gb3 levels (see www.fabrygenphen.com) and often found in patients with cardiovascular disease of likely multifactorial origin (Table 1). Even more challenging from a classification point-of-view are rare missense variants in *GLA*, a common finding upon genetic testing. [5] Even though they may be associated with low GLA activity in leukocytes, and even though Gb3 deposits may be found in, e.g., podocytes and cardiomyocytes, such variants do not necessarily cause disease. Hypomorphic GLA alleles are likely to shift the equilibrium of Gb3 clearance towards the left, but this is no proof of variant pathogenicity. Indeed, it is not even clear to which degree, if any, Gb3 deposits in different tissues are linked to Fabry-related pathology in that tissue. [6]

**Table 1:** Clinical and GLA-related data from six males belonging to a large family segregating the NM_000169.3(*GLA*):c.335G>A, p.(Arg112His) missense variant, that has a general population minor allele frequency (MAF) of 0,001% in the gnomAD database.

ID, YoB, Age at diagnosis/ Age at last follow up	Enzyme activity (leucocytes)	Plasma LysoGb3 (nmol/L), untreat.	Classical symptoms	CV disease risk factors	Clinical presentation	Cardiac status	Renal status	Neurological status	Other
001, 63/63	0.98%	1.9	AP: – AK: – CV: ?	Obesity: - Smoking: – HT: + Dysl: - DM: -	Index patient, renal insufficiency (NB family history: familial renal insufficiency not compatible with X-linked inheritance)	Fibrosis: - CMR mean native T1 value: 966 ms (normal) LVMi: 44 g/m^2^	NTx age 60	WML: + CVA/TIA: -	Kidney biopsy: inconclusive (no electron microscopy)
002, 49/68	2%	1.6	AP:- AK:- CV:-	Obesity: + Smoking: quit HT: + Dysl: + (statin) DM: -	Index patient, neuropathy (NB HMSN phenotype, not-Fabry related)	Fibrosis: - CMR mean native T1 value: T1 between 963 en 984 (normal) LVMi: ~58 g/m^2^ Sinus arrest at age 58, pacemaker at age 63	eGFR >90 mL/min/1,73 m^2^	WML: + CVA 51, TIA 54, 57	Skin biopsy: no zebrabodies
003, 55/71	2.39%	1.9	AP:- AK:- CV:-	Obesity: + Smoking: - HT: + Dysl: + (stat) DM: +	Family screening	Fibrosis: - LMVi: ~45 g/m^2^ Sinus arrest, pacemaker implantation at age 62 Septal hypertrophy	eGFR: 70 mL/min/1,73 m^2^, microalbuminuria	WML: + CVA/TIA: -	Cardiac biopsy: no zebrabodies
004, 53/82	2.73%	2	AP:- AK:- CV:+	Obesity + Smoking: - HT: + Dysl: + (statin) DM: -	Index, renal insufficiency	Fibrosis: + LVMi: ~58 g/m^2^ Multiple cardiac complications starting age 53 Died of cardiovascular disease at age 82 years	Dialysis started age 68 NTx at age 72	WML: + Multiple TIAs starting age 43	Kidney biopsy: zebrabodies present in podocytes, no storage in epithelial cells
005, 40/62	4.55%	1.4	AP:- AK:- CV:-	Obesity: - Smoking: - HT: - Dysl: + (statin) DM: -	Family screening	Fibrosis: - CMR mean native T1 value: 1032 (normal) LVMi: ~59 g/m^2^ No cardiac complications	eGFR: 64 mL/min/1,73 m^2^, microalbuminuria	WML: -	Kidney biopsy: zebrabodies present in podocytes, no storage in epithelial cells Skin biopsy: no zebrabodies
006, 38/62	2.73%	1.2	AP:- AK:- CV:-	Obesity: + Smoking: - HT: + Dysl: + (statin) DM: -	Family screening	Fibrosis: - CMR mean native T1 value: 961 ms (normal) LVMi: ~66 g/m^2^ AF since age 61, CAG age 61 and 62: no significant CAD	eGFR 69 mL/min/1,73 m^2^, microalbuminuria	WML: +	Skin biopsy: no zebrabodies

Two individuals were investigated for Fabry disease because of clinical signs/symptoms that after evaluation and follow-up were deemed not related to the c.335G>A GLA variant (subject 1: renal insufficiency with a non-X linked inheritance pattern, subject 2: neuropathy with a HMSN phenotype). Three individuals with the c.335G>A variant did develop cardiovascular/renal disease, but this always happened in the context of other risk factors (subjects 2/3/4). So the GLA variant may potentially add to the overall CVD risk but does not cause monogenetic disease.

Enzyme activity expressed as % of mean lower and upper reference range.

*AP* acroparesthesia, *AK* angiokeratoma, *CV* cornea verticillata, *HT* hypertension, *Dysl* dyslipidemia, *LVMi* left ventricular mass index, *microalb* microalbumiuria, *WML* white matter lesions, *AF* atrial fibrillation, *CAD* coronary artery disease, *CMR* cardiac MRI, *CMR/lab* values at last assessment at outpatient clinic.

As examples, individuals from two families with low GLA activity, but not Fabry disease, are presented in Table 1 and Supplementary Table S1. Please note that lysosomal Gb3 deposits in podocytes were present in kidney biopsies in both families, a finding that sometimes is taken as proof of Fabry disease.

To give a Fabry diagnosis, the clinical picture and/or family history should be quite Fabry syndrome specific, and in addition the lyso-Gb3 level should be clearly elevated. Clinically, the most specific Fabry findings are cornea verticillate and - if present - multiple angiokeratomas in the lower abdomen and groin. A less specific symptom is acroparesthesias worsened by exercise and hot weather. Unspecific findings are hypertrophic cardiomyopathy, arrythmias, stroke, renal failure, and small fiber polyneuropathy. [7, 8] Low GLA activity due to a missense variant should not be considered as sufficient proof that Fabry disease is the diagnosis, despite being a risk factor for both cardiomyopathy and cardiovascular disease, but not the only (and monogenic) cause of this [9].

We believe that reduced GLA activity associated with genetic variants in the *GLA* gene should be considered to have three possible clinical outcomes: (a) Fabry disease: loss-of-function variants abolishing *GLA* expression or function, (b) GLA insufficiency: major reduced function missense variants that represent a risk factor for cardiovascular disease but not Fabry disease, and (c) hypomorphic alleles: minor reduced function missense variants that are without biochemical or clinical evidence of significant impact. In addition to the clinical picture and family history, the most important distinguishing factor is the lyso-Gb3 level. By making this distinction the correct weight to the identified genetic variant can be given, preventing unnecessary psychological stress as well as ensuring the correct use of Fabry disease specific treatment.

## Supplementary information

Supplementary Table S1

## Data Availability

All data generated or analysed during this study are included in this published article and its supplementary information files.
