# Supplementary material for: GLA insufficiency should not be called Fabry disease
Source: Eur J Hum Genet. 2024 Jun 27;33(3):263–5. doi: 10.1038/s41431-024-01657-0 (PMC11893757; doi:10.1038/s41431-024-01657-0)
Supplement: Supplementary file 1 — Supplementary Table S1 [file 41431_2024_1657_MOESM1_ESM.docx]

**Supplementary Table S1**: Results of investigations of six individuals from the same family carrying the rare missense variant NM_00169.2(*GLA*):c.124A>C, p.(Met42Leu), ascertained after the index patient (Female 57) was diagnosed with focal segmental glomerulosclerosis (FSGS), and a kidney biopsy revealed podocyte Gb3 deposits. Other family members have no kidney or heart disease. Values in bold are considered abnormal. Gen = generation, NA = not assessed. The + and ++ in the renal biopsy column indicate low to moderate degrees of lipid (Gb3) deposits in kidney biopsies.

| Gen | Sex/age | Blood-GLA  (> 2.3 μkat/kg) | U-Gb3 (< 10 μmol per  mol creatinine) | P-Gb3 (< 3.3 μmol/L) | Lyso-Gb3 (< 3.5 nmol/L) | Renal biopsy | Cornea verticillata |
| --- | --- | --- | --- | --- | --- | --- | --- |
| F1 | Female 57 | 11.6 | **14.7** | 2.6 | 1.2 | **+** | Absent |
| F1 | Female 55 | 10.4 | **28.9** | 3.1 | 1.7 | **+** | Absent |
| F2 | Female 36 | 13.3 | 9.4 | 2.3 | NA | **+** | Absent |
| F2 | Male 35 | **1.4** | NA | 2.9 | NA | **++** | Absent |
| F2 | Male 30 | **1.5** | NA | 2.5 | 3.2 | **++** | Absent |
| F3 | Male 8 | **0.8** | NA | NA | **3.8** | NA | NA |
